# Attitude towards informed consent practice in a developing country: a community-based assessment of the role of educational status

**DOI:** 10.1186/1472-6939-15-77

**Published:** 2014-10-22

**Authors:** Kenneth Amaechi Agu, Emmanuel Ikechukwu Obi, Boniface Ikenna Eze, Wilfred Okwudili Okenwa

**Affiliations:** Department of Surgery, University of Teaching Hospital (UNTH), Ituku/Ozalla Enugu, 400001 Nigeria; Department of Community Medicine, UNTH, Ituku/Ozalla, Enugu, Nigeria; Department of Ophthalmology, UNTH, Ituku/Ozalla, Enugu, Nigeria; Department of Surgery, Enugu State University Teaching Hospital Park Lane, Enugu, Nigeria

**Keywords:** Informed consent, Educational status, Attitude

## Abstract

**Background:**

It has been reported by some studies that the desire to be involved in decisions concerning one’s healthcare especially with regard to obtaining informed consent is related to educational status. The purpose of this study, therefore, is to assess the influence of educational status on attitude towards informed consent practice in three south-eastern Nigerian communities.

**Methods:**

Responses from consenting adult participants from three randomly selected communities in Enugu State, southeast Nigeria were obtained using self-/interviewer-administered questionnaire.

**Results:**

There were 2545 respondents (1508 males and 1037 females) with an age range of 18 to 65 years. More than 70% were aged 40 years and below and 28.4% were married. More than 70% of the respondents irrespective of educational status will not leave all decisions about their healthcare to the doctor. A lower proportion of those with no formal education (18.5%) will leave this entire decision-making process in the hands of the doctor compared to those with tertiary education (21.9%). On being informed of all that could go wrong with a procedure, 61.5% of those with no formal education would consider the doctor unsafe and incompetent while 64.2% of those with tertiary education would feel confident about the doctor. More than 85% of those with tertiary education would prefer consent to be obtained by the doctor who will carry out the procedure as against 33.8% of those with no formal education. Approximately 70% of those who had tertiary education indicated that informed consent was necessary for procedures on children, while the greater number of those with primary (64.4%) and no formal education (76.4%) indicated that informed consent was not necessary for procedures on children. Inability to understand the information was the most frequent specific response among those without formal education on why they would leave all the decisions to the doctor.

**Conclusion:**

The study showed that knowledge of the informed consent practice increased with level of educational attainment but most of the participants irrespective of educational status would want to be involved in decisions about their healthcare. This knowledge will be helpful to healthcare providers in obtaining informed consent.

## Background

The World Medical Association Declaration of Lisbon on the rights of the patient has outlined vital components of informed consent. The patient has the right to self determination, a mentally competent adult has the right to give or withhold consent to any diagnostic or therapeutic procedure and has the right to the information necessary to make his/her decisions. Such information should be given in a way appropriate to the patient’s culture and in such a way that the patient can understand. The patient also has the right to explicitly request not to be informed [1]. The doctrine of informed consent emphasizes the right to self-determination of a patient. It requires a physician, before embarking on any diagnostic or therapeutic procedure which carries with it a reasonable possibility of harm to explain the attendant risks of the procedure and its alternatives and to obtain the competent, voluntary and understanding consent of the patient to proceed [2]. There is a distinction between consent obtained in clinical practice (for medical procedure, treatment, or surgery) and informed consent for research which is heavily regulated. The Belmont Report and the Nuremberg Code both address voluntary informed consent as a requirement for the ethical conduct of human subjects research [3, 4]. For consent obtained in clinical practice, patients need care, benefits and risks are better defined, the goal is to benefit individual patients rather than patients in general, and care-givers are more formally trained and licensed. For research on the other hand, in addition to other stringent requirements, the subjects must be made to clearly understand that the consent is for research and not therapy. Also the process of consenting is ongoing and must be made clear to the subject that he or she has the right to withdraw from the study at any time [5]. It has been suggested that the term “informed consent” should be changed to “informed request” for a procedure by the patient thus reflecting the active role the patient plays and to underscore the fact that the patient retains the right to decline consent [6].

In Nigeria the Code of Medical Ethics (Rule 19) deals with the policy of informed consent in terms similar to what is generally obtainable in western countries [7]. Information given to the patient must include the benefits and risks of a procedure, appropriate professional advice on options, the patient’s choice of preferred option and authorization for the clinician to commence treatment by completing a standard form. Consent should be obtained from the patient except in situations where the patient is unable to give consent. Such situations include when dealing with a minor, unconscious patients, accident victims etc. In such cases, consent may be obtained from the next of kin, relations, a court of law or the most senior doctor in the institution. Despite the foregoing the actual observance and implementation of the consenting process in Nigeria is still poor. In a study by Jebbin and Adotey, 74.6% of the patients admitted for surgery were informed of their diagnoses. However, only 36.7% were informed of the possible complications of the surgery [8].

Many studies have been done on this subject and this one looks at the issues on informed consent from the patient perspective with respect to educational status. Although data were collected in the questionnaire for other variables, the authors decided after seeing the results, to focus on educational status to make a strong statement that *the uneducated and poorly educated also want to be informed.* It has also been pointed out that education helps to neutralize the various cultural and social factors that may influence demand for consent and bridges the gap between the doctor and the patient, encouraging discussion on medical matters [9]. The study is community based to avoid the bias that might be introduced interviewing patients in the hospital environment under a care-giver. A study found that participants who were patients were significantly influenced by medical personnel regarding the decision making processes for participation in clinical trials when compared to healthy volunteers [10]. In a previous study in Nigeria only 20% of the doctors who participated regarded obtaining informed consent solely as respect for the patient’s right to self-determination [11]. The rest sought informed consent when dealing with an educated or enlightened patient, when there is a high risk of complications or to avoid malpractice lawsuits [11]. Another study by Irabor and Omonzejele reported similar findings [12]. The American Medical Association has over a period of 144 years moved drastically from overt physician paternalism to greater patient autonomy [13]. Could this be due to the increasing awareness of the patients on their right to know and be involved? It has been reported that the desire for patient autonomy and readiness to seek consent is directly related to educational attainment of the patient [8, 11, 14]. Some studies have, however, shown that there may be underestimation of needs by patients for detailed peri-operative information [15–17]. Some surgeons opt for partial disclosure of information believing that full disclosure of information may affect the patient adversely [18]. In an international survey of breast cancer patients, 63% of the physicians thought that the patients were ‘overwhelmed’ by the amount of information available to them while in fact only 16% of the patients felt ‘overwhelmed’ [19]. The traditional healer in many societies is seen as possessing special and privileged knowledge of the art of healing and the patient is expected to place implicit trust on him or her. An invitation to the patient to participate in the decision making process could be interpreted as lack of competence. This attitude of paternalism emanating from sociocultural factors is assumed to have been carried over to the orthodox physician and some patients would therefore not consider it improper if their wishes are not considered by the attending physician [12]. Poor educational background has been blamed for this passivity [20]. This study attempts to discover the general attitude of the population (from where patients are derived) to the practice of informed consent and the relationship to educational attainment. This information will be of use to doctors especially surgeons in the management of patients.

## Methods

The study was carried out in Enugu, the capital of Enugu State in southeast Nigeria. It is an urban city with a population of approximately 3,972,823 people based on projections from the 2006 population census figures for Nigeria [21]. The state is served by four tertiary health facilities. A list of all the communities/layouts in the 3 Local Government Areas that comprise the state capital viz Enugu South, Enugu North and Enugu East was used as the sampling frame out of which 3 communities were randomly selected. Using convenient sampling a cross-sectional study of all consenting adults was carried out using a semi-structured questionnaire. Ethical permission for the study was obtained from the University of Nigeria Teaching Hospital Ethics Committee.

The questionnaire was critically studied by all the authors and ambiguous, lengthy and otherwise inappropriate questions were removed. The questionnaire was divided into two sections. The first part asked questions on biodata, educational status, occupation etc. while the second part dealt with attitude to various aspects of the informed consent doctrine and practice. A pretest involving 200 participants was carried out in another community similar in demographic variables to the selected communities. The responses were reviewed by the authors before a final version of the questionnaire was produced.

### Study definitions

Participant: any respondent who was either interviewed or completed the questionnaire.

Primary education: Those who completed the first level of formal education after nursery/kindergarten.

Secondary education: Those who completed the second level (post-primary) education.

Tertiary education: Those who completed university or polytechnic or equivalent level of education.

The questionnaires were administered to senior secondary school students (excluding those below 18 years) and their teachers, workers in banks and other offices, artisans, farmers, housewives and unemployed people. Resident doctors from the department of Surgery of the University of Nigeria Teaching Hospital Ituku/Ozalla, Enugu were trained on how to uniformly administer the questionnaires. They subsequently travelled to the selected communities for two to four days spending about three hours each day collecting the data. The educated respondents completed the questionnaires while those without formal education were interviewed in their local language which happened to be the same with the interviewers. The data were analyzed using Statistical Package for Social Sciences (SPSS) version 15. Unanswered questions in the questionnaire were reported as ‘no response’ and were reported as such in the tables. The analysis was descriptive and univariate. Chi-square for trend was used to analyze differences in response from the increasing levels of education. Confidence interval was 95% and P value <0.05 was regarded as significant.

## Results

There were 2545 respondents comprising 1508 males (~ 60%) and 1037 females with an age range of 18–65 years. More than 70% were aged 40 years and below and 723 [28.4%] were married (Table 1 shows details of the demographic distribution of the respondents).

**Table 1:** **Socio-demographic characteristics of 2548 respondents**

Characteristic	n = 2545 [%]
*Age (yrs)*	
<20	1439 [56.4]
21-30	256 [10.0]
31-40	159 [6.3]
41-50	206 [8.2]
51-60	272 [10.7]
61-70	213 [8.4]
*Gender*	
Male	1508 [59.3]
Female	1037 [40.7]
*Marital status*	
Married	723 [28.4]
Single	1680 [66.0]
Widowed	75 [2.9]
Others	37 [1.5]
No response	30 [1.2]
*Educational status*	
University/polytechnic	389 [15.3]
Secondary	1539 [60.5]
Primary	225 [8.8]
No formal education	351 [13.8]
No response	41[1.6]
*Occupation*	
Professional/lecturer	25 [1.0]
Civil servant	118 [4.6]
Teacher (sec & prim)	105 [4.1]
Trading/business	167 [6.6]
Artisan (mason, carp etc.)	221 [8.7]
Student	1352 [53.1]
Unemployed	505 [19.8]
Others	52 [2.0]

As regards consent for medical procedures, only a total of 588 respondents (23.8%) will leave all the decisions to the doctor. The rest (>70%) will want to be involved in such decisions. Level of education did not significantly influence whether or not the person would leave all decisions to the doctor. Only 65 (18.5%) of those with no formal education and 83 (21.9%) of those with university/polytechnic education would leave the doctor to take all decisions about their health management (Table 2). With increasing education, the proportion of those who would require the doctor to disclose everything about the procedure and be allowed to make a choice significantly decreased from 227 [64.7%] in those with no formal education to 213 [56.2%] in those with university/polytechnic education (P < 0.01). Conversely, those with higher education were more likely to require disclosure of only the information the doctor considered necessary as shown in Table 2 (P < 0.01).

**Table 2:** **Decision process for medical procedures**

Response	Educational status					
	University/polytechnic	Secondary	Primary	No formal education	Total	***χ*** ^2^for trend [P value]
	N = 379	N = 1517	N = 225	N = 351	N = 2472	
Will leave all decisions to the doctor	83 [21.9%]	382 [25.2%]	58 [25.8%]	65 [18.5%]	588 [23.8%]	1.689 [0.194]
Will require the doctor to disclose everything about the procedure and be allowed to make a choice	213 [56.2%]	683 [45.0%]	131 [58.2%]	227 [64.7%]	1254 [50.7%]	18.9 [<0.01]*
Will require the doctor to disclose only the information he considers necessary	83 [21.9%]	452 [29.8%]	36 [16.0%]	59 [16.8%]	630 [25.5%]	12.96 [<0.01]*

*Significant.

The authors sought to find out the attitude of the respondents to full disclosure of the possible outcomes and complications of a procedure. The information obtained with respect to educational status is displayed in Table 3. In the sample studied, being informed about all that could go wrong with a procedure would elicit a feeling of confidence in the doctor among 228 (64.2%) participants with tertiary and 801(52.9%) of those with secondary education. However this same action would cause 98 (43.6%) of those with primary and 216 (61.5%) of those with no formal education to consider the doctor unsafe and incompetent. These observed differences were statistically significant (P < 0.01). However the differences in the decision to go to another doctor on account of full disclosure of information among the various levels of education was not statistically significant (Table 3, P = 0.5).

**Table 3:** **Feeling on being informed of all that could go wrong with a procedure**

Response	Educational status					
	University/polytechnic	Secondary	primary	No formal education	Total	***χ*** ^2^for trend [P value]
	N = 355	N = 1515	N = 225	N = 351	N = 2446	
Feel safe and consider the doctor competent	228 [64.2%]	801 [52.9%]	80 [35.6%]	49 [14.0%]	1158 [47.3%]	222.33 [<0.01]*
Feel unsafe and consider the doctor incompetent	28 [7.9%]	219 [14.5%]	98 [43.6%]	216 [61.5%]	561 [22.9%]	421.41 [<0.01]*
Will simply go to another doctor	73 [20.6%]	350 [23.1%]	42 [18.7%]	72 [20.5%]	537 [22.0%]	0.46 (0.50)
Others	26 [7.3%]	145 [9.6%]	5 [2.2%]	14 [4.0%]	190 [7.8%]	8.86 [<0.01]*

*Significant.

An increasing number of those with primary 118 (55.9%), secondary 1132 (77.0%) and tertiary 311(85.7%) education would rather have the doctor who is to carry out a procedure be the one to discuss the procedure and obtain their consent, while 113 (33.8%) of those with no formal education chose to have the discussion and consent obtained from them by a junior doctor. The differences in response across the respondents based on educational status on who obtains their consent (Table 4) is statistically significant (P < 0.01) except for obtaining consent from a nurse where the differences did not reach statistical significance. Four (0.3%) of the respondents indicated that consent could be obtained by any of the listed persons.

**Table 4:** **Whom do you expect should discuss with you and obtain consent?**

Person	Educational status					
	University/polytechnic	Secondary	Primary	No formal education	Total	***χ*** ^2^for trend [P value]
	N = 363	N = 1471	N = 211	N = 334	N = 2379	
Nurse	27 [7.4%]	206 [14.0%]	9 [4.3%]	51 [15.3%]	293 [12.3%]	2.26 [0.13]
Junior doctor	13 [3.6%]	50 [3.4%]	61 [28.9%]	113 [33.8%]	237 [10.0%]	308.47 [<0.01]*
Doctor who does procedure	311 [85.7%]	1132 [77.0%]	118 [55.9%]	113 [33.8]	1674 [70.4%]	289.60 [<0.01]*
Appointee of doctor	12 [3.3%]	79 [5.4%]	23 [10.9%]	57 [17.1]	171 [7.2%]	65.13 [<0.01]*
Any of the above	0	4 [0.3%]	0	0	4 [0.2%]	65.13 [<0.01]*

*Significant.

More, 979 (66.8%) of those with secondary education and 253 (70.5%) of those with tertiary education indicated that informed consent was necessary for procedures on children, while 145 (64.4%) of those with primary and 268 (76.4%) with no formal education indicated that informed consent was not necessary for procedures on children (Table 5). The differences in response were statistically significant (P < 0.01).

**Table 5:** **Is informed consent necessary for children?**

	Educational status				
	University/polytechnic	Secondary	Primary	No formal education	Total
	N = 359	N = 1466	N = 225	N = 351	N = 2401
Yes	253 [70.5%]	979 [66.8%]	80 [35.6%]	83 [23.6%]	1395 [58.1%]
No	106 [29.5%]	487 [33.2%]	145 [64.4%]	268 [76.4%]	1006 [41.9%]

*χ* ^2^ for trend = 250.85, P = < 0.01 [significant].

The acceptance to obtain consent for children is significantly higher with increasing education.

(P < 0.01).

The respondents’ reasons for leaving the decision to the doctor and accept only to be told what the doctor considers necessary is depicted in Table 6. The responses ranged from the ‘philosophical’ (leaving everything to God) to the seemingly ‘logical’ (trusting in the doctor’s knowledge of everything concerning a procedure). Noted however is the fact that confidence in the doctor knowing everything about the procedure was also a more frequent reason indicated among the educated [tertiary, 30 (36.1%); secondary, 80 (20.1%); primary, 14 (24.1%)] as against those with no formal education [7 (10.8%)]. This difference reached statistical significance (P < 0.01). Inability to understand the information was highest among the respondents with no formal education. A significant majority of the respondents in all the education cadres had other reasons outside those provided for leaving the decision to the doctor.

**Table 6:** **Reasons for requiring the doctor to disclose only the information that he considers necessary**

Response	Educational status					
	University/polytechnic	Secondary	Primary	No formal education	Total	***χ*** ^2^for trend [P value]
	N = 83	N = 399	N = 58	N = 65	N = 605	
The doctor knows everything about the procedure	30 [36.1%]	80 [20.1%]	14 [24.1%]	7 [10.8%]	131 [21.7%]	9.03 [<0.01]*
I may not understand the information	2 [2.4%]	21 [5.3%]	0	19 [29.2%]	42 [6.9%]	9.75 [<0.01]*
The doctor may feel offended	0	0	5 [8.6%]	0	5 [0.8%]	6.83 [0.01]*
Seeking such information may appear as lack of trust	0	0	6 [10.3%]	5 [7.7%]	11 [1.8%]	31.11 [<0.01]*
It is better to leave everything in the hands of God	21 [25.3%]	79 [19.8%]	0	3 [4.6%]	103 [17.0%]	19.35 [<0.01]*
Others	30 [36.1%]	219 [54.9%]	33 [56.9%]	31 [47.7%]	313 [51.7%]	1.31 [0.25]

*Significant.

Table 7 shows the reasons for requiring full disclosure and be allowed to make a choice. A comparatively greater proportion of the more educated respondents [tertiary 27 (13%), secondary 171 (24.7%)] understood that it is their right to know all risks, benefits, hazards and alternatives of a procedure to enable them to take a decision than the less educated. However, only 7 (3.1%) of the respondents with no formal education consider it a right to be so informed. This difference is statistically significant (P < 0.01). The other responses are depicted in Table 7.

**Table 7:** **Reasons for requiring full disclosure and be allowed to make a choice**

Reason	Educational attainment					
	University/polytechnic	Secondary	Primary	No formal education	Total	***χ*** ^2^for trend [P value]
	N = 207	N = 693	N = 138	N = 227	N = 1265	
It is my right to know	27 [13.0%]	171 [24.7%]	0	7 [3.1%]	205 [16.2%]	36.34 [<0.01]*
I may sue the doctor or hospital if this is not done	19 [9.2%]	17 [2.5%]	23 [16.7%]	10 [4.4%]	69 [5.5%]	0.21 [0.65]
I am worried about the possible effects of the procedure	10 [4.8%]	53 [7.6%]	9 [6.5%]	16 [7.0%]	88 [7.0%]	0.30 [0.58]
I want to ensure the doctor is competent	4 [1.9%]	14 [2.0%]	8 [5.8%]	26 [11.5%]	52 [4.1%]	36.92 [<0.01]*
It is important for my health insurance policy	5 [2.4%]	46 [6.6%]	5 [3.6%]	9 [4.0%]	65 [5.1%]	0.03 [0.86]
Others	142 [68.6%]	392 [56.6%]	93 [67.4%]	159 [70.0%]	786 [62.1%]	3.43 [0.06]

*Significant.

## Discussion

This study shows that generally more than 75% of all the respondents irrespective of educational status will want to be involved in decisions about their healthcare. However, slightly more than half will want full disclosure of information. In a population survey exploring how much information healthy Japanese would expect from their doctors in the event they developed cancer, 84.5% of the respondents wanted full disclosure [22]. The difference may be due to the higher literacy level in Japan but could also be because the survey in Japan dealt specifically with a widely dreaded disease – breast cancer. This second view may be more plausible seeing that a greater proportion of those without formal education will require the doctor to disclose all the information about the procedure and allow them make their choice than those with secondary and tertiary education. A similar finding has been made previously with respect to drug trials [23]. The reason/s for this apparent departure from the expected attitude is not evident from this study. But the significance is that it will be erroneous to ignore uneducated or poorly educated patients in decision-making about their health on the assumption that they do not desire to be informed. Health practitioners in Nigeria whose duty involves procedures requiring informed consent need to be aware of this while providing care to patients. Being informed about all that could go wrong with a procedure would elicit a feeling of confidence in the doctor among 228 (64.2%) of those with tertiary and 801 (52.9%) of those with secondary education. However this same action will cause 43.6% (98) of those with primary and 61.5% (216) of those without formal education to consider the doctor unsafe and incompetent. This appears contradictory to the responses in Table 2. The ‘all information’ needed to be disclosed might have been understood to refer to ‘positive’ information only. Another focused study may be needed to unravel this. A previous study has also shown that patients with low literacy have difficulty comprehending medical information and physicians instructions and vice versa [24]. Use of simplified materials and information, appropriate for people with lower educational attainment has been shown in previous studies to result in better understanding of medical procedures among patients [25, 26]. The proportion of the participants who would prefer to give consent to the doctor who will carry out the procedure increases with the level of educational attainment. This probably indicates that the better educated respondents consider obtaining informed consent to be more than a routine exercise. The physician who will perform a procedure is not only in a better position to explain the details but is directly responsible to the care receiver. Regarding procedures in children a similar pattern is observed. About 71%, 67%, 36% and 22% of those with tertiary, secondary, primary and no formal education respectively considered obtaining informed consent necessary in children (Table 5).

Surprisingly as seen in Table 6, those with tertiary education were most likely “to leave everything in the hands of God”. From the perspective of European culture, this may be difficult to explain but the strong influence of religion and the high percentage of adherence to Christianity and Islam in Nigeria may hold the key to understanding this attitude [27].

Confidence in the doctor knowing everything about the procedure was also a commoner option among those with tertiary education (36.1%) as against those with no formal education (32.8%). However, a high percentage of the responses were outside the specified options included by the authors in the questionnaire (they came under the option ‘others’). Some of these other responses included but not limited to (a) the doctor is a busy person, (b) I don’t have any questions to ask, (c) I can get more information from the internet etc.

The greater proportion of the more educated respondents understood that it is their right to know all risks, benefits, hazards and alternatives of a procedure to enable them to take a decision than the less educated respondents [tertiary 27 (13%), secondary 171 (24.7%), no formal education 7 (3.1%)] (Table 7). This is to highlight the observation that despite the fact that informed consent is anchored on respect for human rights, the uneducated in the developing world may not view it in that light. However, a greater proportion of all the respondents had other reasons for opting for full disclosure of risks, benefits etc. for informed consent. Some of these reasons included (a) will like to postpone the treatment, (b) need to discuss with friends and relatives, (c) will go for prayers before the procedure and (e) live with the problem out of fear etc.

## Conclusions

The study shows that the desire to be involved in decision-making about respondents’ health is not related to educational status. Therefore, informed consent policy should be conscientiously upheld irrespective of one’s educational attainment. The more educated respondents had better understanding of the doctrine and practice of informed consent policy. Seeing therefore that the uneducated and poorly educated desire to be informed, the instrument for obtaining consent should be simplified to make for easier comprehension.

### Limitations of the study

Non-availability of adequate funds and logistics limited the geographical coverage of the study.

## Authors’ information

KA is a senior lecturer in the Department of Surgery University of Nigeria Enugu Campus and a consultant general surgeon with the University of Nigeria Teaching Hospital Enugu, Nigeria. EI is a senior lecturer in the Department of Community Medicine University of Nigeria Enugu Campus. WO is a senior lecturer and a consultant orthopaedic surgeon at the Enugu State University of Science and Technology Enugu, Nigeria.
